# Causal association between rheumatoid arthritis and an increased risk of age-related macular degeneration: A Mendelian randomization study

**DOI:** 10.1097/MD.0000000000037753

**Published:** 2024-04-12

**Authors:** Mengzhu Zhang, Lincheng Duan, Yue Feng

**Affiliations:** a Chengdu University of Traditional Chinese Medicine, Chengdu, China.

**Keywords:** age-related macular degeneration, rheumatoid arthritis, Mendelian randomization

## Abstract

This study’s goal is to evaluate if there is a causal connection between rheumatoid arthritis (RA) and age-related macular degeneration (AMD), despite past epidemiological studies suggesting an association between the 2 disorders. The impact of RA on AMD is still unknown. Mendelian randomization (MR) was utilized in this study to assess the two-sample causal relationship between RA and AMD. Summary data from GWAS for RA and AMD in individuals with all European ancestries were gathered using the IEU GWAS database. The GWAS summary statistics of RA (14,361 RA patients and 43,923 healthy controls) and AMD (14,034 AMD patients and 91,214 controls participated) were obtained from the IEU GWAS database. After identifying suitable instrumental variables in line with the 3 MR assumptions, we conducted MR using the Mendelian randomization-Egger (MR-Egger), weighted median, and inverse variance weighting techniques. The MR-Egger intercept and MR-Polyvalent Residuals and Outliers methods were used to investigate the effects of horizontal pleiotropy. The leave-one-out strategy was used to prevent bias caused by certain single nucleotide polymorphisms. Sensitivity analysis was used to detect the heterogeneity. Using 50 single nucleotide polymorphisms as instrumental variables, this study examined the relationship between RA and AMD and discovered that RA increased the risk of AMD (inverse variance weighting odds ratio [OR] = 1.056, 95% confidence interval [CI] = 1.02–1.09, *P* = 5.44E−04; weighted median OR = 1.085, 95% CI = 1.04–1.14, *P* = 4.05E−04; MR-Egger OR = 1.074, 95% CI = 1.01–1.14, *P* = 2.18E−2). The current investigation demonstrated a causal link between AMD and RA. RA increased the risk of AMD. It is advised that future research concentrate on the processes underlying the relationship between RA and AMD.

## 1. Introduction

Systemic autoimmune illness rheumatoid arthritis (RA) is characterized by inflammatory cell infiltration and aberrant fibroblast-like synovial cell growth.^[1]^ It is characterized by tendon inflammation that causes bone erosion and cartilage loss.^[2]^ impacting 1% of the population.^[3]^ Globally, there are regional variations in the frequency of RA, with industrialized nations typically having a greater prevalence. This may be because of exposure to environmental risk factors, but it might also be because of genetic variables, differing demography, and underreporting in other regions of the world.^[4]^ While RA prevalence has grown during the past 3 decades, RA severity has decreased.^[5]^ This is a significant obstacle for the world healthcare system.

Macular degeneration is the most prevalent cause of visual loss in people over 60, and its prevalence increases with age.^[6]^ Due to the aging population, a rise in instances is anticipated. With the number of new cases rapidly rising each year, the direct cost of age-related macular degeneration (AMD) to the North American healthcare system topped $250 billion in 2008.^[7]^ Poor eyesight can lead to a higher risk of falling, depression, and the need for long-term care if it makes it difficult to do everyday tasks like getting dressed, eating, and working.^[8]^ For Europeans, AMD continues to be a serious public health issue.^[9]^ Rheumatoid arthritis is closely associated with many diseases. Some Mendelian randomization studies suggest a causal relationship between RA and osteoporosis,^[10]^another subset of studies showed that RA is not causally related to osteoporosis, and that osteoporosis in RA patients may be associated with side effects of antirheumatic therapy and reduced physical activity.^[11]^ Previous studies have found the positive effects of rheumatoid arthritis on inflammatory bowel disease.^[12]^ However, it may play a role in the development of several specific cancers, such as cervical cancer and pancreatic cancer.^[13]^ The risk of hypothyroidism increases with increasing RA, and hypothyroidism is associated with an increased risk of RA.^[14]^

Both illnesses are brought on by a confluence of hereditary and environmental risk factors. Activation of the alternate pathway (AP) of the complement system is a common factor in the development of AMD and RA. Smoking, prolonged sun exposure, and genes linked to the disorder are among the environmental and genetic risk factors for macular degeneration. In AMD, factor H can directly activate AP if it is not suppressed. One estimate places the hereditary component of RA’s heterogeneity at 60% of the disease’s susceptibility.^[15]^ Antibodies to citrullinated proteins are closely associated with RA and are a risk factor for severe joint deterioration since they may be detected years before symptoms develop.^[16]^ Recent epidemiological studies demonstrate that RA and AMD are becoming more prevalent in non-European populations. Additionally, the AP of complement determines how pathogenic these antibodies are.^[17]^ New genetic variables linked to the underlying illnesses of RA and AMD have been discovered by several investigations.^[18]^ However, this does not adequately explain their connection. Several studies have alleged a connection between the 2 illnesses.^[19]^ Some research claims there is no connection between the 2 illnesses.^[20]^ Previous studies have disputed whether there is a relationship between RA and AMD, and there is no evidence of a causal relationship between the 2 diseases.

Confounding factors and reverse causation are drawbacks of conventional epidemiological investigations.^[21]^ Due to the possible bias of confounding factors, these previous observations are restricted to association inference; even this result is debatable. However, public health or medical activities focused on exposure would be futile and a waste of resources if there was no causal relationship between exposure and outcome. Randomized controlled trials are time- and money-consuming, even if they may be able to establish a link between RA and AMD.^[22]^ Mendelian randomization (MR) tested this hypothesis by examining if the association between risk factors and outcomes could be accounted for by causal effects.^[23]^ Mendel law of “random assignment of parental alleles to offspring” was used to replicate random assignment in MR, just as it would be in a randomized controlled experiment.^[24]^ The confounding issues posed by observational research and reverse causality can be avoided by genetic polymorphism and allelic randomization, which offer superior support for causal inference than conventional observational studies.^[25]^ To investigate whether RA and AMD are causally connected in the present study, we used a two-sample MR analysis.

## 2. Methods

### 2.1. Overview of the study design

**Figure 1.**
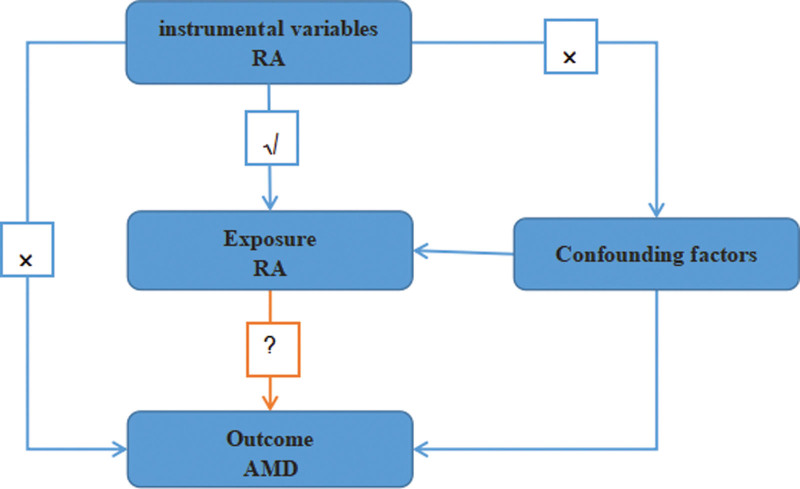
Schematic diagram. Mendelian randomization (MR) study’s two-sample design. RA, rheumatoid arthritis; AMD, age-related macular degeneration. The “×” denotes that instrumental variables cannot directly affect outcome or are not related with Confounding factors, but rather through the exposure route. The “√” denotes a strong correlation between instrumental variables and exposure.

We measure the causal effects using a two-sample MR method. In a nutshell, we calculate the relationship between RA and AMD. Selection of relevant genetic instrumental variables (IVs) for the associated exposures, application of various MR approaches, evaluation of pleiotropic effects, and heterogeneity and sensitivity analysis were the 3 crucial phases of the study. The schematic is shown in Figure 1.

### 2.2. Data source

To eliminate the impacts of population stratification, all single nucleotide polymorphisms (SNPs) and their associated pooled data were collected from studies that only included populations with European ancestry. It is possible to download the IEU genome-wide association studies (GWAS) database, which includes GWAS summary statistics for RA, at https://gwas.mrcieu.ac.uk/datasets/. For data analysis, those of European ancestry were included. 14,361 individuals with RA and 43,923 healthy controls were enrolled in the research (GWAS ID: ebi-a-GCST002318). Additionally, we searched the IEU GWAS database for any publicly available GWAS on AMD. 14,034 AMD patients and 91,214 controls participated in this research (GWAS ID: ebi-a-GCST010723). All RA cases met the 1987 American College of Rheumatology criteria for RA diagnosis,^[26]^ or were diagnosed with RA by a professional rheumatologist. The RA cases included in the GWAS meta-analysis were obtained from 14,361 RA cases and 43,923 controls from 18 European studies. A meta-analysis of AMD-related GWAS was obtained from 11 data sources,^[27]^ including the International AMD Genomics Consortium and the UK Biobank and color fundus photography of early AMD phenotypes. Additionally, we calculated the F statistic for the accumulation of SNPs using the formula shown below: F = (N-k-1)R2/k(1-R2). R2 represents the change in exposure that each IV explains. The strength of the instrument was assessed using the F statistic (IVs with F values below 10 were considered weak).

All information was gathered from previously released research. Therefore, neither patient permission nor ethical approval was necessary for this investigation.

### 2.3. Selection of instrumental variables

As IVs in MR, genetic variations were exploited. To examine the impact of IVs on exposure and outcome, researchers employed 2 different samples. They were assisted in their study by publicly accessible data from a variety of GWAS. Several requirements have to be completed to acquire objective findings.^[28]^ Here are a few of these things to consider: (1) IVs are statistically significantly connected with exposure; (2) confounding variables related to exposure, result, or outcome, particularly outcome, should not be associated with IVs; and (3) IVs can only affect the outcome through exposure. SNPs was chosen as the IV following the 3 MR theories.

To test the first assumption, we first identified SNPs across the genome that were associated with exposure and were statistically significant (*P* < 5E−8). These SNPs also showed some potential for mutation (small allele frequencies; MAF > 5%). To remove the SNP connected to considerable linkage disequilibrium, we utilized a clustering approach with R2 0.001 and a 10,000 kb window size.

Second, we looked for SNPs linked to confounding factors and outcomes (*P* < 5E−8) in all SNPs connected with exposures in the Phenoscanner database repository (http://www.phenoscanner.medschl.cam.ac.uk/phenoscanner). To support the presumption that genetic instrumental factors are independent of outcomes and confounding variables, these SNPs were eliminated. In addition, we also employed the MR-Polyvalent Residuals and Outliers approach to reduce outliers. Palindromic SNPs with moderate allele frequencies were excluded when coordinating the GWAS data for RA and AMD.

### 2.4. Sensitivity analysis

All analyses were performed using the R software version 4.0.3 and the packages “TwoSampleMR”version 0.5.6 and “MRPRESSO”version 1.0.

Utilizing pleiotropy, heterogeneity, and sensitivity analyses, quality control was carried out. To determine if there was horizontal pleiotropy, we employed MR-Egger regression. Additionally, MR-Polyvalent Residuals and Outliers experiments were run to assess whether pleiotropy existed,^[29]^ and outlier SNPs were deleted manually. Heterogeneity was assessed using the inverse variance weighting (IVW) approach and Egger regression, and it was quantified using Cochran Q statistic.^[30]^ The results were recalculated by removing each unique SNP one at a time to ensure the validity of the findings. We also used the leave-one-out method to remove some SNPs that had negative effects on the findings and then recalculate the results. We screened in the Phenoscanner database (http://www.phenoscanner.medschl.cam.ac.uk/) to eliminate SNPs unrelated to exposure factors and to only retain all SNPs related to exposure.

### 2.5. Mendelian randomization analysis

All analyses were performed using the R software version 4.0.3 and the packages “TwoSampleMR”version 0.5.6 and “MRPRESSO”version 1.0.

We used a range of MR methods to validate the causal relationship between RA and AMD, with Mendelian randomization-Egger (MR-Egger), weighted median (WM), and IVW approaches predominating. According to the heterogeneity selection effect model, there was no heterogeneity in this study, and the IVW method chose the fixed effect model. The IVW approach accurately determines the causal relationship between exposure and outcome and has the highest statistical validity.^[31]^ Egger test nevertheless produced a trustworthy estimate of the causal impact and a valid test of the null causal hypothesis, even if all genetic variants were incorrectly instrumented.^[32]^ The use of WM approaches can result in reliable estimations when some genetic instruments are not real instrumental factors.^[33]^Unpredictable results The IVW technique was used to combine the causal effects of individual SNPs. The odds ratio (OR) and 95% confidence interval (CI) evaluated the relative risk associated with the target illness.

## 3. Results

Following strict exclusion criteria, we included 50 SNPs for RA. IVs with f-statistics > 10, indicating no weak instrumental variable bias. We did not find heterogeneity using Cochran Q test. (*P* > .05). The IVW method was used for the fixed effects model. The correlation between AMD and RA was shown to be statistically significant (IVW OR = 1.056, 95% CI = 1.02–1.09, *P* = 5.44E−04; WM OR = 1.085, 95% CI = 1.04–1.14, *P* = 4.05E−04; MR-Egger OR = 1.074, 95% CI = 1.01–1.14, *P* = 2.18E−2). See Table 1 for details. MR-Egger regression showed no horizontal pleiotropy (P for MR-Egger intercept > 0.05). More figures in supplementary material are provided for scatter plots (Fig. S1, Supplemental Digital Content, http://links.lww.com/MD/M137), funnel plots (Fig. S2, Supplemental Digital Content, http://links.lww.com/MD/M139), forest plots (Fig. S3, Supplemental Digital Content, http://links.lww.com/MD/M141), and leave-one-out plots (Fig. S4, Supplemental Digital Content, http://links.lww.com/MD/M142).

**Table 1:** MR estimates of RA on AMD.

Exposure	Outcome	Method	OR	95% CI	*P*	F
RA	AMD	IVW	1.056	1.024–1.089	5.44E−4	35.45
		MR-Egger	1.074	1.012–1.139	2.18E−2	
		WM	1.085	1.037–1.135	4.05E−4	

AMD = age-related macular degeneration, CI = confidence interval, IVW = inverse variance weighted, MR = Mendelian randomization, MR-Egger = Mendelian randomization-Egger, OR = odds ratio, RA = rheumatoid arthritis, WM = weighted median

## 4. Discussion

The current study carefully examined the cause-and-effect relationship between RA and AMD using integrated GWAS data. To our knowledge, this is the first study to use MR methods to investigate the relationship between RA and AMD. The results of this two-sample MR investigation point to a connection between AMD and RA. According to our research, RA raises the likelihood of developing AMD. According to earlier research, those with RA are more likely to develop new-onset AMD. According to these findings, the current investigation found that RA was a risk factor for AMD. We contrasted the results of the current investigation with those of a recent retrospective study.^[34]^ We evaluated how RA affected the onset of AMD. The retrospective study discovered that those with RA had a greater risk of getting new-onset AMD. RA individuals received an AMD diagnosis sooner than controls (*P* .0001). RA is among the risk factors for AMD. However, this retrospective study was not able to completely exclude the effect of confounding factors. In our investigation, RA was also identified as an AMD risk factor. (IVW OR = 1.056, 95% CI = 1.02–1.09, *P* = 5.44E−04; WM OR = 1.085, 95% CI = 1.04–1.14, *P* = 4.05E−04; MR-Egger OR = 1.074, 95% CI = 1.01–1.14, *P* = 2.18E−2). We discovered that the findings on the impact of RA on AMD were in line with retrospective clinical observational studies, we discovered. AMD risk factors include RA. In contrast to other research, our inquiry revealed a causal link between RA and AMD, and our analysis revealed that RA was a risk factor for AMD. The existence of a causal link, in contrast to epidemiological studies, may imply that the detection rate of AMD among RA patients in our study is far from reality or that RA treatment strategies are effective in preventing AMD.

In RA and AMD, the genetic contribution is a key factor in family aggregation. The genetic loci connected with human leukocyte antigen (HLA) loci enhance susceptibility to RA, whereas those related to adenosine triphosphate-binding cassette margins increase susceptibility to AMD. However, these genetic loci do not account for the majority of instances of either illness. Rheumatoid arthritis is a genetic and environmental condition that is influenced by a combination of factors. The likelihood of getting RA is genetically predisposed to an extent of about 60%.^[35]^ In several populations, genome-wide association research found more than 100 loci associated with RA risk.^[36]^ The HLA gene region is a genetic risk factor for RA. Gene expression levels for HLA-A and HLA-B are associated with RA. Some of the susceptibility genes of RA may affect RA by regulating gene expression at the protein level.

Although neither illness is predominantly influenced by environmental factors, environmental influences, including lifestyle choices and drug usage, cannot be discounted. For instance, the research found that patients using active thyroxine supplements following a thyroid hormone imbalance had a greater chance of developing AMD.^[37]^ However, there is no evidence linking a thyroid hormone imbalance to the onset of RA. Another study indicated that smoking was associated with an elevated risk of disease and that heavy smoking was more significantly associated with this risk.^[38]^ Significant inflammatory alterations were seen in RA. AMD is recognized to have a greater risk of inflammation.^[8,39]^ This may assist in illuminating why RA may be a risk for AMD.

The complex pathogenesis of AMD includes genetic, age, inflammatory, environmental, and lifestyle variables such as smoking, nutrition, obesity, and alcohol use.^[40]^ According to one study, AMD is linked to more than 100 uncommon mutations in addition to 52 recognized common variants. and this research offers a thorough justification for the genetic predisposition to AMD in Europeans. The elevated risk allele, the protective allele, and the most discriminating risk allele between patients with advanced AMD and control participants were all found in ARMS2, respectively. The ACE Alu+/+ genotype and the ApoE4 allele have both been proven to be protective against AMD. The majority of patients have several genetic risk pathways, which points to a complicated etiology for AMD. The most reliable primary risk factor for AMD is age.

Both RA and AMD have intricate genetic and environmental etiologies that are unknown. According to reports, smoking increases the likelihood of developing RA and AMD.^[41,42]^ The aryl hydrocarbon receptor (AhR), a transcription factor that is abundantly expressed in a variety of tissues and cells, notably immune cells, is regarded as a key participant in the immune response. Both smoking and nonsmoking patients’ human RA peripheral blood mononuclear cells were found to express AhR and its downstream genes, with smoking patients showing considerably increased expression.^[43]^ Smoking may influence the AhR pathway, which is involved in the onset of RA. It was discovered that participants who were current smokers (*P* .001) progressed to advanced AMD at a younger age (3.9 and 1.7 years, respectively) in comparison to the reference group of never-smokers. For smokers who are currently smoking, altering their habit (i.e., becoming a former smoker) would have stopped around 60% of the progression to late-stage AMD. By improving their behavior, the entire research population’s 14.7% illness rate may have been avoided.^[44]^ The existence of the same genetic pattern may help explain the correlation found in observational research. The current investigation discovered a causal link between RA and AMD even after accounting for comparable genes and confounding factors.

Mendelian randomization experiments have supported the causal link between RA and AMD. RA increased the risk of AMD. Mendelian randomization studies have the advantage of being able to effectively avoid the influence of various confounding factors on association results, which is challenging to do in observational studies. In addition, the level of evidence in the analysis results is higher than that of observational studies. The publicly accessible GWAS summary statistics form the basis of MR. Without raising the price of experiments, it is possible to harvest reliable genetic data. The validity of the genetic instrumental factors was thoroughly checked for positivity, which also increased the plausibility of the findings of the later study that RA and AMD are causally associated. There is support for the logic of routine vision examination in RA patients. A detrimental causal link between RA and AMD was shown using MR. As a result, the author advises increased visual acuity screening for RA patients with caution.

There are several restrictions on this study. The minimal number of samples for RA and AMD might produce false positive and negative findings. The statistical power of MR analysis is restricted by the few genetic loci that have so far been identified in GWAS due to sample size restrictions. To boost the power of testing connections, a larger GWAS with a larger sample size and SNP are necessary to replicate the MR results. RA and AMD are both widespread illnesses with rising frequency in populations of non-European ancestry, even though we chose data from a cohort with only European ancestry. Recent epidemiological research shows that non-European populations are developing new cases of RA and AMD.^[5,45]^ Although this causal association was shown by steady data, the OR of the current study was low. The present study has a flaw in that it did not stratify patients with RA and AMD to investigate the relationship between RA and dry AMD and wet AMD, respectively.

Additionally, communities differ in terms of environmental triggers and genetic vulnerability. Future research on populations with various lifestyles should therefore aim to replicate findings on environmental and genetic risk factors in populations with various geographic regions, age stages, work environments, and cultural practices, as well as identify novel correlations that may be specifically linked to a particular population. Other races also require MR investigations, and these studies require extensive GWAS data to be validated.

In conclusion, AMD and RA are related causally. RA increased the risk of AMD. For RA patients, routine visual acuity screening is advised. Future research on RA and AMD could focus on the mechanisms by which RA affects AMD.

## Acknowledgments

The studies or consortiums cited in this study and used in it are gratefully acknowledged by the authors for contributing open datasets.

## Author contributions

**Data curation:** Lincheng Duan.

**Writing – review & editing:** Yue Feng.

**Writing – original draft:** Mengzhu Zhang.

## Supplementary Material








