# Supplementary material for: Causal association between rheumatoid arthritis and an increased risk of age-related macular degeneration: A Mendelian randomization study
Source: Medicine (Baltimore). 2024 Apr 12;103(15):e37753. doi: 10.1097/MD.0000000000037753 (PMC11018156; doi:10.1097/MD.0000000000037753)
Supplement: Supplementary file 1 [file medi-103-e37753-s001.docx]

**Supplementary Figure 1.**

**Scatter plot**


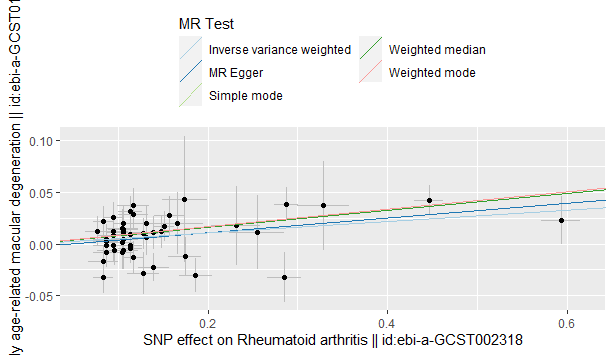
*The slope coefficient offers an estimate of the bias of the causal impact, and the estimate of intercept may be read as an estimate of the average pleiotropy of all single-nucleotide polymorphisms (SNPs).*
