# Supplementary material for: Causal association between rheumatoid arthritis and an increased risk of age-related macular degeneration: A Mendelian randomization study
Source: Medicine (Baltimore). 2024 Apr 12;103(15):e37753. doi: 10.1097/MD.0000000000037753 (PMC11018156; doi:10.1097/MD.0000000000037753)
Supplement: Supplementary file 2 [file medi-103-e37753-s002.docx]

**Supplementary Figure 2.**

**Funnel plot**


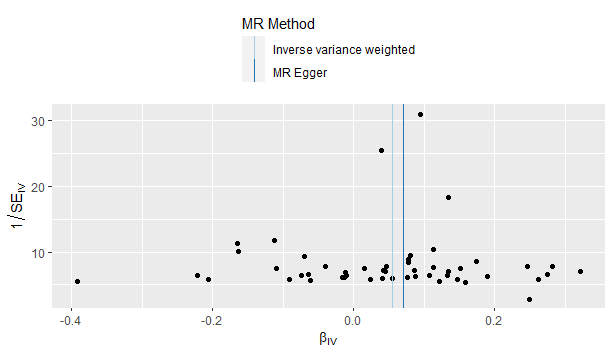


*RA on AMD.The x-axis represents β, and the y-axis represents 1/SE (standard error).*
