# Supplementary figures and images for: Causal association between rheumatoid arthritis and an increased risk of age-related macular degeneration: A Mendelian randomization study
Source: Medicine (Baltimore). 2024 Apr 12;103(15):e37753. doi: 10.1097/MD.0000000000037753 (PMC11018156; doi:10.1097/MD.0000000000037753)

**Supplementary Figure 3.**

**Forest plot (MR effect size for RA on AMD)**


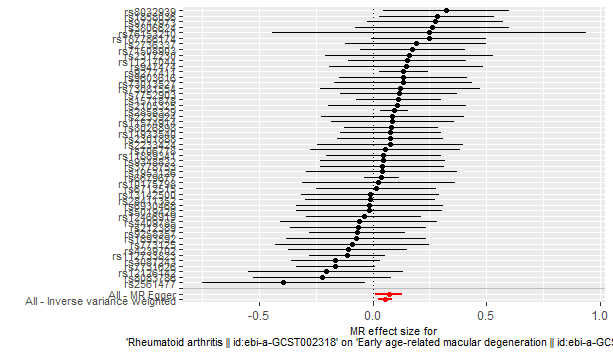

Supplement: Supplementary file 3 [file medi-103-e37753-s003.docx]

**Supplementary Figure 4.**

**MR leave-one-out sensitivity analysis for RA on AMD**


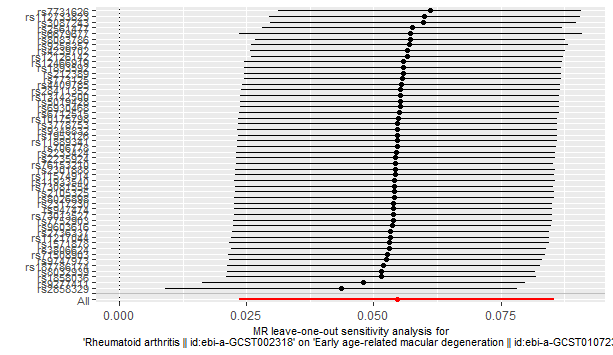

Supplement: Supplementary file 4 [file medi-103-e37753-s004.docx]
